# Assessing the Feasibility of Typhoid Elimination

**DOI:** 10.1093/cid/ciaa585

**Published:** 2020-07-29

**Authors:** Jeffrey D Stanaway, Phionah L Atuhebwe, Stephen P Luby, John A Crump

**Affiliations:** 1 Institute for Health Metrics and Evaluation, University of Washington, Seattle, Washington, USA; 2 World Health Organization, Regional Office for Africa, Brazzaville, Congo; 3 Infectious Diseases and Geographic Medicine, Stanford University, Stanford, California, USA; 4 Centre for International Health, University of Otago, Dunedin, New Zealand

**Keywords:** typhoid, *Salmonella* Typhi, elimination, control, vaccine

## Abstract

In 1993, the International Task Force on Disease Eradication classified the political will for typhoid eradication as “none.” Here we revisit the Task Force’s assessment in light of developments in typhoid vaccines and increasing antimicrobial resistance in *Salmonella* Typhi that have served to increase interest in typhoid elimination. Considering the requisite biological and technical factors for elimination, effective interventions exist for typhoid, and humans are the organism’s only known reservoir. Improvements in water supply, sanitation, hygiene, and food safety are critical for robust long-term typhoid control, and the recent Strategic Advisory Group of Experts on Immunization recommendation and World Health Organization prequalification should make typhoid conjugate vaccine more accessible and affordable in low-income countries, which will allow the vaccine to offer a critical bridge to quickly reduce burden. While these developments are encouraging, all current typhoid diagnostics are inadequate, having either poor performance characteristics, limited scalability, or both. No clear solution exists, and this should be viewed as a critical challenge to any elimination effort. Moreover, asymptomatic carriers and limited data and surveillance remain major challenges, and countries considering elimination campaigns will need to develop strategies to identify high-risk populations and to monitor progress over time. Finally, policymakers must be realistic in planning, learn from the planning failures of previous elimination and eradication efforts, and expect unforeseeable shocks and setbacks. In the end, if we assume neither unanticipated breakthroughs in typhoid control nor any chaotic shocks, history suggests that we should expect typhoid elimination to take decades.

Typhoid fever is a bacteremic febrile illness caused by systemic infection with *Salmonella enterica* serovar Typhi (*Salmonella* Typhi). Recent estimates range from 11 to 21 million illnesses annually [1–4], resulting in 117 000 (95% uncertainty interval [UI], 65 000–188 000) deaths [4] and 8.4 million (95% UI, 4.7–13.6 million) disability-adjusted life years (DALYs) [4]. Burden is greatest in countries with poor water supply and sanitation, especially those in South Asia, Southeast Asia, and sub-Saharan Africa. In 2017, the World Health Organization (WHO) Strategic Advisory Group of Experts (SAGE) on Immunization recommended typhoid conjugate vaccines (TCVs) in endemic regions for infants and children 6 months and older, and adolescents and adults up to 45 years of age [5]. In the same year the WHO prequalified the TCV, giving typhoid-endemic, low-income countries access to and, through Gavi, the Vaccine Alliance (Gavi), funding for typhoid vaccine [6]. These developments, combined with growing antimicrobial resistance in *Salmonella* Typhi, have led to increased interest in the possibility of eliminating typhoid.

In 1997, the Dahlem Workshop on the Eradication of Infectious Diseases established definitions for disease control, elimination, eradication, and extinction [7]. Here, it is useful to first distinguish the *elimination of a disease* from the *elimination of infections*: While the former refers to a reduction to zero of typhoid fever disease incidence in a defined geographic area through deliberate efforts, the latter refers to reducing to zero both disease incidence and the number of infections from *Salmonella* Typhi. The Dahlem Workshop group defined *control* as the reduction of disease to a locally acceptable level through deliberate efforts. Subsequently, the term *elimination of a disease “as a public health problem”* has been popularized as an alternative term for control using the same definition, but perhaps with the advantage of greater appeal to policymakers. *Eradication* extends the concept of elimination of infections from one or more geographic areas to the globe: the permanent reduction to zero of incidence of infection worldwide. Finally, *extinction* includes that the infectious agent no longer exists in nature or the laboratory. Unlike control and elimination, eradication and extinction provide the advantage that the intervention or interventions are no longer required.

In 1993, the International Task Force on Disease Eradication screened 94 infectious diseases to assess their potential eradicability [8]. They classified 28 of these into 3 categories: (1) diseases targeted for eradication (cysticercosis, dracunculiasis, lymphatic filariasis, mumps, poliomyelitis, rubella); (2) diseases/conditions of which aspects could be eliminated (hepatitis B, iodine deficiency disorders, neonatal tetanus, onchocerciasis, rabies, trachoma, yaws, and other endemic treponematoses); and (3) diseases not eradicable now or not eradicable (n = 15). The Task Force gave the remaining 66 diseases limited consideration, including typhoid, for which they classified the political will for eradication as “none.”

Here we revisit the Task Force’s assessment in light of developments in typhoid vaccines and increasing antimicrobial resistance in *Salmonella* Typhi that have served to increase interest in typhoid elimination. We review considerations for typhoid elimination, including biological, technical, economic, social, and political factors, and discuss the role of vaccination in an elimination effort, data gaps that could undermine a potential elimination effort, and a possible timeline for typhoid elimination.

## BIOLOGICAL AND TECHNICAL FACTORS

Three broad biological and technical factors are necessary before disease elimination can be considered feasible: (1) an effective intervention is available to interrupt transmission of the agent; (2) practical diagnostic tools with sufficient sensitivity and specificity are available to detect levels of infection that can lead to transmission; and (3) humans are essential for the life cycle of the agent, which has no vertebrate reservoir and does not amplify in the environment.

Several effective interventions are available to interrupt typhoid transmission. Improvements in water supply, sanitation, and hygiene (WASH) and food safety practices have proven adequate to effectively eliminate typhoid in high-income countries of Europe, North America, and elsewhere. In addition to effectively interrupting typhoid transmission, improvements in water supply and sanitation are part of the Sustainable Development Goals sixth goal; they have the additional benefit of reducing the risk of other water and foodborne infections, and thus offer broad socioeconomic benefits [9]. While the large-scale centralized 19th-century approaches to WASH seen in most high-income countries may be difficult and expensive to implement in 21st-century low-income city conditions, alternative approaches are being developed. For example, passive point of collection disinfection technologies exist that are compatible with existing intermittent supply systems, and are low-cost and easy to use. Such systems do not require strong municipal-level governance, can use existing shared water points, and demand minimal behavior change.

Parenteral unconjugated Vi polysaccharide vaccine and the oral live-attenuated Ty21a vaccine have been available for many years and since 2008 have been recommended for typhoid control [10], but their use has been limited in endemic areas. The reasons for limited use of older typhoid vaccines in endemic countries are likely many, and the lack of protection of infants and young children precluded adoption into routine immunization schedules. TCVs overcome this limitation and are also likely to provide a longer duration of protection. While we can have a high degree of confidence that TCV can contribute to reduction of typhoid disease as a component of elimination, we have less information about their potential role in eliminating infection. Few data are available on the effects of typhoid vaccines on fecal shedding of *Salmonella* Typhi, and studies on the indirect effects of TCV for herd protection are yet to be reported. Disease modeling efforts suggest that typhoid elimination is unlikely to be achieved by vaccination alone, although TCVs are likely to have a greater impact than older typhoid vaccines [11]. A key uncertainty in such models is the role of chronic carriers of *Salmonella* Typhi [11], known to play a major role in non-travel-associated typhoid transmission in high-income, low-incidence settings.

The clinical presentation of typhoid is not pathognomonic and laboratory tests are essential to accurately establish a typhoid diagnosis. While commonly used, the Widal test and other serologic approaches have unreliable test characteristics that limit their usefulness as surveillance tools. Blood culture has high specificity, but low sensitivity [12], and requires laboratory facilities and expertise that make it expensive and difficult to scale. Bone marrow culture has higher sensitivity than blood culture, but its cost and invasiveness make it impractical at scale [13]. With that, the limitations of all current typhoid diagnostics present a challenge and no clear solution exists to the need for an accurate and scalable typhoid diagnostic. Despite being a critical component of a disease elimination strategy, the detection of *Salmonella* Typhi infection in the absence of disease poses even greater challenges. *Salmonella* Typhi may be shed in the absence of disease [14], and acute, convalescent, and chronic shedding may follow disease. Like blood culture, stool culture lacks sensitivity for identifying shedding, and although specificity is high, repeat stool culture is likely impractical at scale. We know that Vi serology may be a useful screening test for chronic carriage in low-incidence settings [15], but the test has performed less well in endemic areas and may be confounded by vaccines that induce an anti-Vi antibody response [16]. Shedding in the absence of disease and chronic carriage represents a potentially large and difficult-to-detect reservoir of bacteria, similar to that of the silent reservoir that confounded historic schistosomiasis elimination efforts [7].

The final biological and technical question is whether humans are the only reservoir of *Salmonella* Typhi. The reservoir is defined as the habitat in which the agent normally lives, grows, and multiplies [17], often referred to as the site of “amplification” of the pathogen. Several lines of evidence are reassuring that humans are the only reservoir of *Salmonella* Typhi. These include the experience of detailed epidemiologic investigations in Europe, North America, and elsewhere, where human chronic carriers become the residual reservoir of autochthonous *Salmonella* Typhi transmission as typhoid incidence approaches zero [18]. Furthermore, there are numerous studies documenting the finite environmental survival of *Salmonella* Typhi outside of the human host in many matrices [19]. However, past elimination and eradication efforts have been thwarted by incorrect assumptions about the pathogen reservoir, notably the unexpected role of symbionts in cholera and dogs in dracunculiasis, so caution is warranted around such assumptions [7].

## ECONOMIC, SOCIAL, AND POLITICAL CRITERIA

The economic considerations for typhoid elimination are numerous, and include competing priorities both within and outside the health sector. For typhoid elimination, there are obvious synergies with other infections controlled by safe water, food, and improved sanitation. However, *Salmonella* Typhi is only one of several *Salmonella* serovars that causes serious infections in humans and for which multivalent vaccines with wider health impact could strengthen the case for investment [20].

Aylward et al stated that “of the lessons learned in the past 85 years, none is more important than the recognition that societal and political considerations ultimately determine the success of a disease eradication effort” [21]. The Dahlem Workshop group identified social and political criteria for disease elimination and eradication, highlighting that societal and political commitment is required from beginning to end and that there is an enormous cost of failure both in economic terms, but also for future efforts. Buy-in is required from all levels of society, including affected communities, and the reasons for elimination or eradication must be robust and accepted widely. There needs to be a broad consensus on priority and justification and political commitment at the highest levels, including at the level of national leaders and respected public figures who are willing to serve as champions. Achieving these criteria requires a multilevel advocacy plan. A technically feasible strategy must be identified, field-tested in a defined area, and found effective. There are many complexities in implementation, and agile programs that can respond to unexpected challenges are more likely to succeed. The effort should lead to a sustainable improvement in health ideally also beyond the disease of primary focus, and ideally the program would have a high benefit-to-cost ratio. Consequences for concurrent campaigns, both for disease elimination and other efforts, need to be considered as well.

## DEMONSTRATION PROJECT

Identifying a technically feasible elimination strategy, field-testing it in a defined area, and demonstrating it is effective are considered critical steps before wider implementation of an elimination effort [7]. Due to their small size and relative isolation, the islands of Oceania have been leaders in efforts to control or eliminate a range of endemic neglected tropical diseases, including lymphatic filariasis, trachoma, yaws, soil-transmitted helminths, leprosy, and scabies. Furthermore, some nations, such as Fiji and Samoa, have both well-documented major typhoid problems, and social and political will to address the disease [22, 23]. The selection of interventions for elimination for field testing would likely include typhoid vaccine, improvements to water and sanitation, and if epidemiologically important, identification and treatment of chronic carriers of *Salmonella* Typhi.

## DATA GAPS AS OBSTACLES TO ELIMINATION

Elimination efforts require data to (1) accurately estimate burden to persuade stakeholders and motivate political will; (2) characterize the spatial distribution of the disease to understand where to prioritize interventions; (3) track progress toward elimination; (4) determine if and when elimination targets have been achieved; and (5) detect control failures.

Since 2000, data on typhoid incidence have become more plentiful, thanks in part to multicenter studies like the Diseases of the Most Impoverished program [24] and the Typhoid Fever Surveillance in Africa Program (TSAP) [25]. With currently ongoing multicenter studies (eg, the Surveillance for Enteric Fever in Asia Project [SEAP] [26], Severe Typhoid in Africa [SETA] program [27], the Strategic Typhoid Alliance Across Africa and Asia [STRATAA] study [28], and Surveillance of Enteric Fever in India [SEFI] [29]), we expect data availability to continue to improve. Still, although data on typhoid incidence have improved, data abundance for typhoid trails behind other causes considered targets for elimination/eradication efforts, and most countries lack systematic typhoid surveillance systems. With that, estimates of typhoid burden, and trends in that burden, continue to largely be model-based predictions. Given the diagnostic challenges discussed previously, and the additional challenges associated with surveillance of acute infections (ie, the short window for case detection precludes survey-based surveillance), data limitations are likely to remain a challenge facing any typhoid elimination effort.

Several studies have produced global estimates of typhoid burden. Estimates from these studies largely agree with regard to global typhoid incidence [4]. Unfortunately, region- and country-level estimates are more heterogeneous. While all studies have estimated moderate to high burden in South Asia, Southeast Asia, and most of sub-Saharan Africa, estimates for these regions, and the countries within them, may differ by an order of magnitude between studies, and country-level policymakers may be reluctant to pursue elimination without first having a clearer sense of the burden in their country. Still, for countries like India, Bangladesh, and Pakistan, who experienced 67% of all global DALYs for typhoid in 2017 [4], the evidence is likely adequate to motivate political will for an elimination strategy and justify large-scale vaccination campaigns. The lack of detailed subnational data on typhoid incidence will likely preclude targeted risk-based approaches to elimination efforts, generally, and vaccination campaigns, specifically.

Any elimination effort would require substantial typhoid surveillance to monitor progress, determine if and when elimination targets are reached, and detect control failures. The current typhoid surveillance infrastructure, to the extent that it exists at all, is inadequate. Given the challenges associated with establishing surveillance systems in low-resource settings, and the cost, expertise, and equipment associated with blood culture, large-scale national surveillance systems are likely infeasible in most typhoid-endemic countries, which will necessitate alternative approaches. SEFI was initiated in 2017 and will use a hybrid system to integrate data collected from active fever surveillance cohorts with hospital and laboratory data to provide timely estimates of typhoid incidence and trends in India [29]. This program may offer a model for typhoid surveillance that could be applied in other endemic countries, and sites from previous studies of typhoid incidence (eg, SEAP, SETA, and TSAP) should be considered as sentinel surveillance sites so as to leverage the existing capacity and expertise. Decision-makers may also pursue other low-cost alternatives to traditional surveillance; to the extent that environmental surveillance proves a useful surrogate measure of human disease, it may offer one such option.

## THE ROLE OF VACCINATION TOWARD TYPHOID ELIMINATION

Typhoid is related to economic development, and improved WASH remains the mainstay for typhoid control. Viewing the history of typhoid in high-income countries, we see that typhoid largely disappeared with improvements in WASH that occurred early in the 20th century. It is, therefore, possible that economic development, and the resulting improvements in WASH, might result in the steady disappearance of typhoid without any effort to target typhoid specifically. Still, improving access to potable water, sanitation and sewage collection, and treatment will take investments and time. Given the large burden of disease and growing antimicrobial resistance, typhoid vaccine can serve as a bridge while countries improve their WASH infrastructures. As such, the vaccine will remain a critical tool for typhoid control in the foreseeable future.

While typhoid vaccine remains an important tool, there are limitations to a vaccine-only strategy that underlie the importance of continued commitment to improvements in WASH. Even in the most robust vaccination campaigns, not all individuals will be vaccinated and, given imperfect vaccine efficacy, protection among those who are vaccinated will be less than 100%. Over time, we can expect vaccination to result in reduced burden. As annual costs of vaccine programs stabilize and burden declines, the cost-effectiveness of immunization will erode, and this may reduce local political support for these investments. Given this potential for declining cost effectiveness, and competing interests, governments are likely to see vaccination as a narrow strategy and prefer a broader approach.

Through the SAGE, WHO has developed policies for TCV use aiding the provision of guidance on decision-making for vaccine introduction either for response to outbreaks or into routine immunization. Zimbabwe was the first African country to use typhoid vaccine as a means of curbing a typhoid outbreak in the midst of a cholera outbreak in the country’s capital city of Harare. Typhoid fever is endemic in Harare, with seasonal outbreaks every year since 2010 due to persistent poor WASH conditions in overcrowded suburbs, a situation not likely to change in the near future. Coupled with a high level of antimicrobial resistance, the National Immunization Technical Advisory Group recommended a mass vaccination campaign with Typbar-TCV, which yielded an optimal administrative coverage (85.4%) in 8 days. TCV was well accepted by communities with no serious adverse events following immunization reported. School vaccination was effective in reaching 90% of the age group 5–15 years.

## TIMELINE FOR TYPHOID ELIMINATION

**Figure 1.**
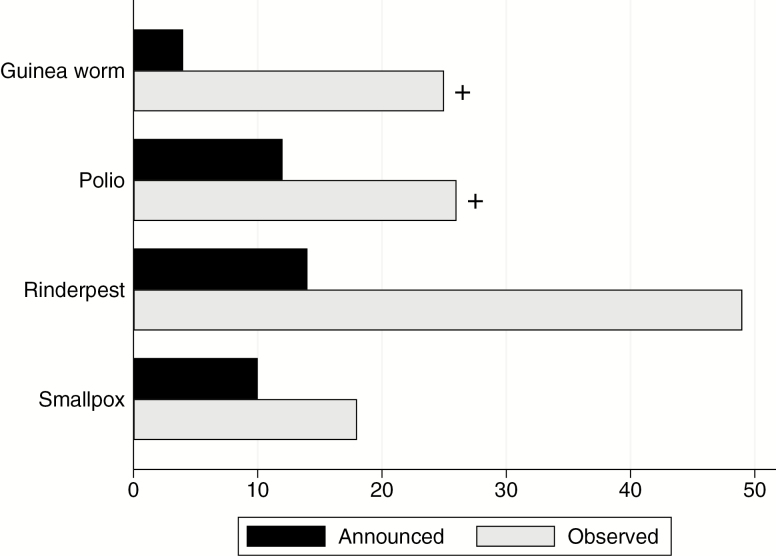
Announced versus observed eradication timelines for Guinea worm, polio, rinderpest, and smallpox. The “+” signs indicate that Guinea worm and polio eradication efforts are ongoing.

The Bill & Melinda Gates Foundation (BMGF) laid out a timeline for typhoid control that calls for effective TCVs with WHO prequalification, SAGE recommendations, and Gavi funding by 2020, with sustained typhoid reduction by 2035 [30]. And while progress is mostly on track to meet the BMGF 2020 goals, the 2035 target remains ambitious. Sustained reduction is a less ambitious goal than complete eradication, but the lessons of eradication efforts remain instructive. Timelines for these eradication efforts have historically been optimistic: Smallpox eradication took 8 years longer than the originally announced timeline of 10 years [31]; rinderpest eradication took 35 years longer than the originally announced timeline of 14 years; Guinea worm eradication was originally planned to take 4 years, and remains incomplete after 25 years [32]; similarly, polio eradication was originally planned to take 12 years, and remains incomplete after 26 years (Figure 1) [33].

The consistently optimistic disease eradication timelines are an example of the planning fallacy, which occurs when plans are based on best-case scenarios and when planners ignore the experience of similar projects (“this won’t happen to us”). The planning fallacy is not unique to disease elimination plans. For example, the plan to construct the Brooklyn Bridge projected a cost of US$5 million and a timeline of 5 years for construction. In the end, construction of the bridge required 12 years and US$15 million [34]. Similarly, while construction of the new Scottish Parliament building in Edinburgh, Scotland, was expected to cost less than £40 million, the final costs were £431 million [35]. In every such case, we see that there are many ways for a plan to fail, that most means of failure are too improbable to anticipate, and that the likelihood of something going wrong on a large project is high.

In addition to challenges in developing and implementing interventions, we must consider the potential for shocks that could interrupt progress. Examples of profound historical shocks include World Wars I and II, the global economic depression of 1929–1939, and the 1815 eruption of Mount Tambora that resulted in the “year without a summer” and the global crop failures of 1816. While shocks are often unpredictable events, existing and emerging challenges suggest possible shocks that could interrupt progress in typhoid elimination: Climate change, population growth in developing countries, declining per capita water availability, and stressed aquifers are examples. Because typhoid has a carrier state, shocks that interrupt control efforts would allow for reintroduction of the disease in areas where control had been achieved, and projections that assume no global shocks are likely to be optimistic.

If we assume neither unanticipated breakthroughs in typhoid control nor any chaotic shocks, history suggests that we should expect elimination to take decades. Breakthroughs in typhoid control technologies, political will, or unexpectedly rapid economic development in typhoid-endemic countries could accelerate elimination, while shocks could delay progress. Improvements in case management that result in low case severity and fatality could reduce political will and extend the time to elimination. Conversely, increasing drug resistance could result in increased disease severity and mortality and, in turn, political will, thereby sparking dramatic action toward elimination.

## WHERE DO WE STAND?

Effective interventions, the lack of any known nonhuman reservoir, and growing political will suggest that eliminating typhoid as a public health problem may be feasible. Still, poor diagnostics, asymptomatic shedding and carriage, and limited data and surveillance remain major challenges. While widespread vaccination should be pursued as a means to quickly reduce burden, especially in the face of growing antimicrobial resistance, improvements in WASH are likely to offer more robust long-term benefits, and we believe that policymakers should develop comprehensive multisectoral plans to control typhoid and other waterborne and fecal–oral diseases. Along with these interventions, improved surveillance for typhoid fever and evidence generation on disease burden and hotspots are needed to support TCV introduction and monitor trends in incidence to support any elimination effort. While none of these challenges are likely insurmountable, policymakers should be clear-eyed about the potential for shocks and planning failures.

## Notes

***Supplement sponsorship.*** This supplement is funded with support from the Coalition against Typhoid Secretariat, housed at the Sabin Vaccine Institute in Washington, DC and made possible by a grant from the Bill & Melinda Gates Foundation.

***Potential conflicts of interest.*** The authors: No reported conflicts of interest. All authors have submitted the ICMJE Form for Disclosure of Potential Conflicts of Interest.
